# Molecular detection of plasmid mediated *bla*_TEM_, *bla*_CTX−M,_and *bla*_SHV_ genes in Extended Spectrum β-Lactamase (ESBL) *Escherichia coli* from clinical samples

**DOI:** 10.1186/s12941-023-00584-0

**Published:** 2023-05-05

**Authors:** Mahesh Kumar Chaudhary, Indrani Jadhav, Megha Raj Banjara

**Affiliations:** 1 School of Life and Basic Sciences, Jaipur National University, Jaipur, India; 2 Department of Microbiology, Nepal Mediciti Hospital, Lalitpur, Nepal; 3 Central Department of Microbiology, Tribhuvan University, Kirtipur, Nepal

**Keywords:** *Escherichia coli*, Extended spectrum β-lactamase, Multi-drug resistance

## Abstract

**Background:**

Extended spectrum β-lactamases (ESBLs) are a group of beta-lactamase enzymes that confer resistance to the oxyimino-cephalosporins and monobactams. The emergence of ESBL - producing genes possesses a serious threat for treating infections since it is associated with multi-drug resistance. This study was focused to identify the ESBLs producing genes from *Escherichia coli* isolates from clinical samples from a referral-level tertiary care hospital in Lalitpur.

**Methods:**

This was a cross-sectional study conducted from September 2018 to April 2020 at the Microbiology Laboratory of Nepal Mediciti Hospital. Clinical samples were processed, and culture isolates were identified and characterized following standard microbiological techniques. An antibiotic susceptibility test was performed by a modified Kirby-Bauer disc diffusion method as recommended by Clinical and Laboratory Standard Institute guidelines.Extended -spectrum beta-lactamases were phenotypically confirmed by the combined disc method. The ESBL-producing genes *bla*_TEM_, *bla*_CTX−M_ and *bla*_SHV_ were confirmed by PCR.

**Results:**

Of the 1449 total *E. coli* isolates, 22.29% (323/1449) isolates were multi-drug resistant (MDR). Among the total MDR *E. coli* isolates, 66.56% (215/323) were ESBL producers. The maximum number of ESBL *E. coli* was isolated from urine 90.23% (194) followed by sputum 5.58% (12), swab 2.32% (5), pus 0.93% (2), and blood 0.93% (2). The antibiotic susceptibility pattern of ESBL *E. coli* producers showed the highest sensitivity toward tigecycline (100%) followed by polymyxin b, colistin and meropenem. Out of 215 phenotypically confirmed ESBL *E. coli*, only 86.51% (186) isolates were found to be positive by PCR for either *bla*_TEM_ or *bla*_CTX−M_ genes. Among the ESBL genotypes, the most common were *bla*_TEM_ 63.4% (118) followed by *bla*_CTX−M_ 36.6% (68).

**Conclusion:**

The emergence of MDR and ESBL – producing *E. coli* isolates with high antibiotic – resistant rates to commonly used antibiotics and increased predominance of major gene types *bla*_TEM_ is a serious concern to the clinicians and microbiologists. Periodic monitoring of antibiotic susceptibility and associated genes would help guide the rationale use of antibiotics for treating the predominant pathogen *E. coli* in the hospitals and healthcare facilities of the communities.

**Supplementary Information:**

The online version contains supplementary material available at 10.1186/s12941-023-00584-0.

## Introduction

Extended-spectrum beta-lactamases (ESBLs) are the group of beta-lactamase enzymes, that hydrolyze and cause resistance to the oxyimino-cephalosporins (cefotaxime, ceftazidime, ceftriaxone, cefuroxime and cefepime) and monobactams (aztreonam), but not the cephamycins (cefoxitin and cefotetan) or carbapenems (imipenem, meropenem, and ertapenem), produced by *Escherichia coli* and *Klebsiella pneumoniae* [1].

The emergence of resistant bacteria worldwide is a threat to favorable outcomes of treatment of common infections in community and hospital settings. *E. coli* is one of the commonest pathogens to exhibit multi-drug resistance. Important risk factors for infection with MDR and ESBL *E. coli* are prolonged antibiotic exposure, overstay in hospital, increased use of third-generation cephalosporins, severe illness, increased use of intravenous devices or catheters [2].

ESBL was first detected during 1983–1990 in different countries [3]. Distinct epidemic clones with TEM and SHV enzymes have been found in Europe, including SHV-12, CTX-M-9, CTX-M-3 andCTX-M-15 [4].

The prevalence of ESBL - producing organisms is more than 20% in Asia and South Africa [5].

In Nepal, also, due to the increasing incidence of ESBL - producing *E. coli*, the cost associated with the treatment has increased. The detection of major genes such as *bla*_TEM_, *bla*_CTX−M_ and *bla*_SHV_ in ESBL producing *E. coli* by molecular methods and their antibiotic resistance pattern can provide valuable information about their epidemiology and help in the formulation of rational antimicrobial therapy [6]. Therefore, this study was conducted with the objectives of determining the spectrum of MDR and ESBL *E. coli* producing strains and molecular characterization of these resistant genes. Characterization of ESBL *E. coli* at the molecular level would be useful for developing better treatment strategy and prevention of the disease.

## Materials and methods

### Sample Processing and identification of Bacteria

A cross-sectional study was conducted in the Microbiology Laboratory of Nepal Mediciti Hospital, Bhaisepati, Nepal from September 2018 to April 2020. Ethical approval was obtained from the Ethical Review Board of Nepal Health Research Council (NHRC), Kathmandu, Nepal. A total of 16,542 clinical samples sent to the microbiology laboratory were processed and cultured following standard microbiological techniques. Bacterial isolates were identified by cultural and morphological characteristics, Gram stain and biochemical tests (triple sugar iron, indole, citrate, urease and motility).

### Antibiotic susceptibility tests

Antibiotic susceptibility testing was performed by a modified Kirby-Bauer disc diffusion method as recommended by the Clinical and Laboratory Standard Institute guidelines. The antibiotics used were amikacin (30 µg), gentamicin (10 µg), ciprofloxacin (30 µg), ceftriaxone (30 µg), cefotaxime (30 µg), ceftazidime (30 µg), nitrofurantoin (300 µg), norfloxacin (10 µg), nalidixic acid (30 µg) ofloxacin (5 µg), cotrimoxazole (25 µg),cefixime (5 µg), cefepime (30 µg), tigecycline (15 µg), imipenem (10 µg), meropenem (10 µg), polymyxin b (300 µg), and colistin (10 µg). Plates were incubated aerobically at 37 °C for 24 h. Zone diameter in millimeters was measured and organisms were identified as sensitive, resistant, and intermediate as per CLSI 2022 guidelines [7]. *E. coli* strain ATCC 25,922 was used as a control strain.

### Screening of ESBL

The screening for ESBL was done by the disk diffusion technique using 3rd generation cephalosporins (ceftazidime, cefotaxime and ceftriaxone). Isolates resistant to more than one of these agents were identified as possible ESBL producers [7].

### Confirmation of ESBL

For confirmation of ESBL, a combined disc test was performed using ceftazidime (30 µg) alone and ceftazidime with clavulanic acid (30 µg/10µg) and cefotaxime (30 µg) and cefotaxime with clavulanic acid (30 µg/10µg). A difference in the zone of inhibition by ≥ 5 mm of either of ceftazidime clavulanic acid with ceftazidime alone and cefotaxime clavulanic acid with cefotaxime alone was interpreted as confirmed ESBL [7].

### Gene detection

From confirmed ESBL *E. coli*, plasmid DNA was extracted using an alkaline hydrolysis method. These plasmid DNAs were used as a template for PCR amplification using *bla*_TEM_, *bla*_CTX−M_ and *bla*_SHV_ specific primers (Marcogen, Korea). For PCR amplification, 1.5 µl plasmid DNA was added to 25 µl mixture containing 13 µl master mixture (Solis Biodyne, Estonia), 10.5 µl nuclease-free water, and 0.5 µl each reverse and forward primers. PCR was performed in 5 Prime/02 thermal cycler using optimized conditions (Bibby Scientific, U.K.). For *bla*_TEM_ gene identification, initial denaturation at 94^o^C for 5 min followed by 30 cycles of each of denaturation (95^o^C for 45 s), annealing (50^o^C for 45 s), and extension (72^o^C for 30 s), and final extension (72^o^C for 10 min). For *bla*_SHV_ and *bla*_CTX−M_ genes, initial denaturation at 94^o^C for 5 min followed by 30 cycles of each denaturation at 95^o^C for 45 s, annealing at 56^o^C for 45 s and 62^o^C for 45 s respectively, and extension at 72^o^C for 30 s, and final extension at 72^o^C for 10 min. The amplified product was subjected to gel electrophoresis (2% gel stained with ethidium bromide) at 70 V for 45 min. A DNA ladder of 100 bp was used to estimate the molecular weight of the amplified products.

### Control of the phenotypic tests and PCR

For the ESBL test, *E. coli* (ATCC 25,922) and *Klebsiella pneumoniae* (ATCC 700,603) were taken as negative control and positive control respectively. Confirmed *E. coli* strains harboring *bla*_TEM_, *bla*_SHV,_ and *bla*_CTX−M_ were taken as positive control and nuclease-free water as negative control.

### Statistical analysis

Data were entered and percentage calculations were analyzed using the Statistical Package for Social Science (SPSS) version 21.

## Results

1449 *E. coli* isolates were recovered from various clinical samples. The highest number of *E. coli* was isolated from urine followed by sputum, swab, pus, blood, fluid, foley’s tip,vaginal swab, catheter tip, BAL, biopsy, bile suction tube, CVP tip, and ET tube. Of the 1449 total *E. coli* isolates, 22.29% (323/1449) isolates were multi-drug resistant. Among the total MDR *E. coli* isolates, 66.56% (215/323) isolates were ESBL producers. The maximum number of ESBL *E. coli* was isolated from urine 90.23% (194), followed by sputum 5.58% (12), swab 2.32% (5), pus 0.93% (2), and blood 0.93% (2) (Table 1).

**Table 1:** Distribution of ESBL *E. coli* from clinical samples

Specimen	% (No)
Urine	90.23% (194)
Sputum	5.58% (12)
Swab	2.32% (5)
Pus	0.93% (2)
Blood	0.93% (2)
Total	100% (215)

**Table 2:** Antibiotic susceptibility pattern of ESBL *E. coli*

Antibiotics	Antibiotic susceptibility of ESBL *E. coli* (n = 215)	
	Sensitive % (No)	Resistant % (No)
Amikacin(AK)	91.6% (197)	8.4% (18)
Gentamicin(G)	83.7% (180)	16.3% (35)
Ciprofloxacin(CIP)	58.2% (125)	41.8% (90)
Ceftriaxone(CTR)	0.0 (0)	100% (215)
Cefotaxime(CTX)	2.7% (1)	97.3% (214)
Ceftazidime(CAZ)	0.0 (0)	100% (215)
Nitrofurantoin(NIT)*	93.8% (182)	6.2% (12)
Norfloxacin(NX)*	56.2% (109)	43.8% (85)
Nalidixic acid(NA)*	4.6% (9)	95.4% (185)
Ofloxacin(OF)*	46.9% (91)	53.1% (103)
Tigecycline(TGC)	100% (215)	0.0 (0)
Imipenem(IPM)	68.8% (148)	31.2% (67)
Meropenem(MRP)	90.2% (194)	9.8% (21)
Polymyxin B(PB)	100% (215)	0.0 (0)
Colistin(CL)	100% (215)	0.0 (0)

* Used in urinary isolates.

The antibiotic susceptibility pattern of ESBL producing *E. coli* showed the highest sensitivity toward tigecycline (100%) followed by polymyxin b, colistin, nitrofurantoin, amikacin, meropenem,gentamicin, imipenem, ciprofloxacin, norfloxacin, and ofloxacin (Table 2).

**Fig. 1:**
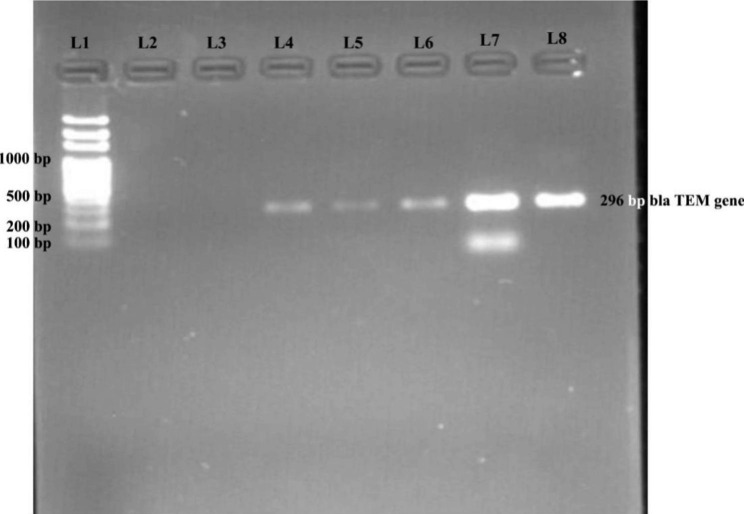
Detection of *bla*_TEM_ genes, cropped image of the gel **Lane 1**:100 bp DNA ladder.**Lane 2**: Negative control, **Lane 8: Positive control, Lane 4–7**: Test plasmids positive for *bla*_TEM_ gene, **Lane 3**: Test plasmid negative for *bla*_TEM_ gene.

**Fig. 2:**
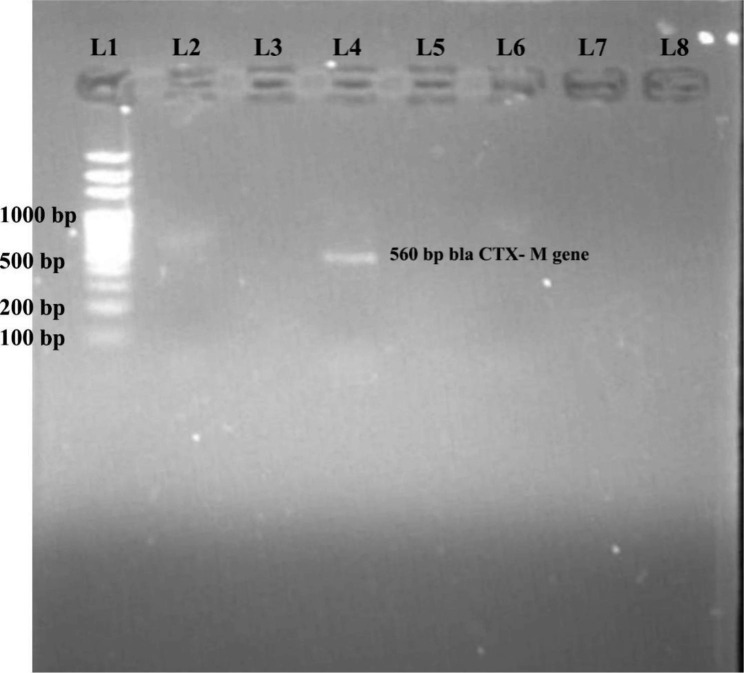
Detection of *bla*_CTX−M_ gene, cropped image of the gel **Lane 1**: 100 bp DNA ladder, **Lane 4: Positive** control, **Lane 3**: Negative control, **Lane 2**: Test plasmid positive for *bla*_CTX−M_ gene, **Lane 5–8**: Test plasmid negative for bla_CTX−M_ gene.

Out of 215 phenotypically confirmed ESBL *E. coli*, only 86.51% (186) isolates were found to be positive by PCR using *bla*_TEM_, *bla*_CTX−M_ and *bla*_SHV_ specific primers (Table 3). 13.49% (29) were negative for any resistant gene tested. Among the ESBL genotypes, the most common were *bla*_TEM_ 63.4% (118) followed by *bla*_CTX−M_ 36.6% (68) (Figs. 1 and 2). The co-existence of *bla*_TEM_ and *bla*_CTX−M_ in ESBL -producing *E. coli* was 20.96% (39). No ESBL *E. coli* isolates co-harbored *bla*_SHV_ and *bla*_TEM_, *bla*_CTX−M_ and *bla*_SHV_ genes.

**Table 3:** Distribution of ESBL genotypes in *E. coli*

ESBL genotypes	% (No) (n = 186)
*bla*_TEM_	63.4% (118)
*bla*_CTX−M_	36.6% (68)
*bla*_TEM_ + *bla*_CTX−M_	20.96% (39)
*bla*_SHV_	0.0 (0)

## Discussion

Despite the discovery of antibiotics, the emergence of MDR and ESBLs producing bacteria due to the extensive use of extended spectrum cephalosporins (ESCs) since the early 1980s is a significant evolution in antimicrobial resistance [8]. Several other factors, including misuse of drugs, inappropriate antibiotic treatment, and extensive use of antimicrobials have also contributed to the emergence of drug-resistant bacteria. This study was conducted in the department of microbiology laboratory, Nepal Mediciti Hospital during a period of September 2018 to April 2020 with the aim of understanding the antibiotic susceptibility profile of MDR and ESBL -producing *E. coli.*

It was found that the highest number of *E. coli* isolates was recovered from urine .With regard to urinary tract infection, *E. coli* showed great extent of resistance to nalidixic acid, co-trimoxazole and third generation cephalosporins. A similar pattern of resistance in urinary isolates of *E. coli* was shown in Nepal [9, 10]. In contrast to our results, Fanta et al. reported 73% *E. coli* isolates were ceftriaxone-resistant [11]. This may be due to the irrational use of third-generation cephalosporins [12]. However, a significant degree of susceptibility was found to nitrofurantoin followed by amikacin and gentamicin. Similar findings have been reported in various studies [13–15]. This may be due to the rational use of these drugs in urinary tract infections (UTIs) cases since nitrofurantoin is reserved drug for UTIs.

In this study, analysis of the antibiotic susceptibility of *E. coli* isolated from sputum, blood, swab, and pus demonstrated a significant degree of susceptibility toward tigecycline (100%) followed by colistin (98–100%), polymyxin b (97–100%), meropenem (91–96%), and imipenem (79–90%). Similar results are shown in other studies [16, 17]. It was found, a higher resistant pattern toward cephalosporins (22%to 93%), fluoroquinolones (26–85%), and aminoglycosides (8–59%) compared to urine isolates. Several studies conducted in Nepal have showed similar results [14, 18]. In contrast to our study, Kubone et al. noted a higher susceptibility pattern toward cephalosporins, fluoroquinolones, and aminoglycosides [19]. The increased level of drug resistance is a major concern worldwide since these are the first-line drugs recommended internationally [20] and are irrationally used in the public and private sectors [21].

This study noted 22.29% (323/1449) MDR *E. coli* isolates that were suspected of being ESBL producers were confirmed by the combined disc method. The prevalence of ESBL *E. coli* was 66.56% (215/323), which was alarmingly high. Several studies reported high prevalence i.e.40–70% of ESBL *E. coli* among MDR *E. coli* [10, 15, 17, 22, 23]. However, the study by Onyedibe et al. in 2018 observed only 18.6% ESBL *E. coli* [24], which is an analogous result to other study [25]. This is not similar to our study due to the variation in geography, study design, and selection of the type of antimicrobial agents. The indiscriminate use of beta-lactam antibiotics leads to the generation of selective pressures, which have led to the selection of various mutated forms of beta lactamase [26]. The antibiotic profiles of ESBL *E. coli* was found to be higher sensitivity toward tigecycline (100%), polymyxin b (100%), colistin (100%) followed by amikacin (91.6%), meropenem (90.2%), and imipenem (68.8%). The susceptibility to nitrofurantoin was 93.8% against ESBL *E. coli* isolated from urine. So, it could be the drug of choice for treating infection caused by ESBL producing *E. coli* similar to the previous studies conducted in India [27, 28].

In this study, out of 323 MDR *E. coli* isolates, ESBL *E. coli* phenotypes were found to be positive in 66.56% (215) isolates. Similar findings were reported by Ozcakar et al. (2011) and Dalela et al. (2012) [29, 30]. 13.49% (29) phenotypic ESBL - positive *E. coli* isolates lacked *bla*_TEM_, *bla*_CTX−M_ and *bla*_SHV_ genes. That could be false - positive results by phenotypic methods or can be the possible presence of other ESBL encoding genes such as SFO, BES, BEL, TLA, GES, PER and VEB types and structural changes in penicillin-binding proteins that result in resistance to β-lactam antibiotics [29, 31–33].

In this study, the overall prevalence of ESBL genes was 86.51% (186), which is similar to other findings reported by Dirar et al., 2020 in Sudan and Ahmad et al., 2019 in Iraq [34, 35]. PCR revealed *bla*_TEM_, *bla*_CTX−M_ and *bla*_SHV_ genes in ESBL - producing *E. coli* were 63.4% (118), 36.6% (68), and 0.0 (0) respectively. In this study, *bla*_TEM_ was the most predominant genotype of ESBL among *E. coli* isolates. Similar findings were reported by Dirar et al., 2020 in Sudan, Ahmad et al.,2019 in Iraq, Noha et al., 2020 in upper Egypt, Pandit et al., 2020 in Nepal, Michael et al., 2018 in Iraq, Sahoo et al. 2019 in India, and Jena et al., 2017 in India [34–40].

In this study, the prevalence of *bla*_CTX−M_ genes was found to be 36.6% (68), which concurs with various reports demonstrating the extensive worldwide dissemination of *bla*_CTX−M_ genes in ESBL - producing *E. coli* isolates [41]. However, another study from Nepal has reported the high prevalence of *bla*_CTX−M_ genes (100%) by Lohani et al., 2019 and (91.4%) by Parajuli et al., 2016 [42, 43].

The differences in frequencies of the prevalence of these genes may be because of differences in time by which isolates were collected and differences in volume and type of antibiotic consumption [44].

Furthermore, multiple harboring of genes in a single ESBL - producing *E. coli* were also noted. The most common combination gene was *bla*_TEM_ + *bla*_CTX−M_ type 20.96% (39). Our finding is in agreement with the study by Lohani et al., where (21.2%) of *bla*_TEM_ and *bla*_CTX−M_ genes were reported [42]. The presence of multiple genotypes in a single isolate might be the result of a complex antibiotic resistance pattern [45].

In this study, *bla*_SHV_ type *E. coli* was not detected, similar to the study in Nigeria [46]. However, several findings in Nepal reported the prevalence of *bla*_SHV_ gene at low frequency [42, 43].

## Conclusion

This study highlights the emergence of MDR and ESBL -producing *E. coli* isolates with high antibiotic -resistant rates to commonly used antibiotics and increased predominance of major gene types *bla*_TEM_. No resistance was documented to tigecycline, polymyxin b, and colistin suggesting the suitable drug of choice for treating ESBL -producing *E. coli* infections. Periodic molecular detection and identification of ESBL -producing bacterial isolates could inform the rationale use of antibiotics to preserve antibiotics for the future.

## Electronic supplementary material

Below is the link to the electronic supplementary material.

Supplementary Material 1

Supplementary Material 2

Supplementary Material 3

## Data Availability

All data generated in the study have been included in the manuscript.

## List of Abbreviations

ESBL: Extended Spectrum Beta Lactamase
MDR: Multi Drug Resistant
bla: β-lactamase-coding gene
ATCC: American Type Culture Collection
CLSI: Clinical Laboratory Standard Institute
CTX-M: Cefotaximase, Munich
TEM: Temoniera gene
SHV: Sulfhydryl variable
UTIs: Urinary Tract Infections
