# Supplementary figures and images for: Molecular detection of plasmid mediated blaTEM, blaCTX−M,and blaSHV genes in Extended Spectrum β-Lactamase (ESBL) Escherichia coli from clinical samples
Source: Ann Clin Microbiol Antimicrob. 2023 May 5;22:33. doi: 10.1186/s12941-023-00584-0 (PMC10163748; doi:10.1186/s12941-023-00584-0)

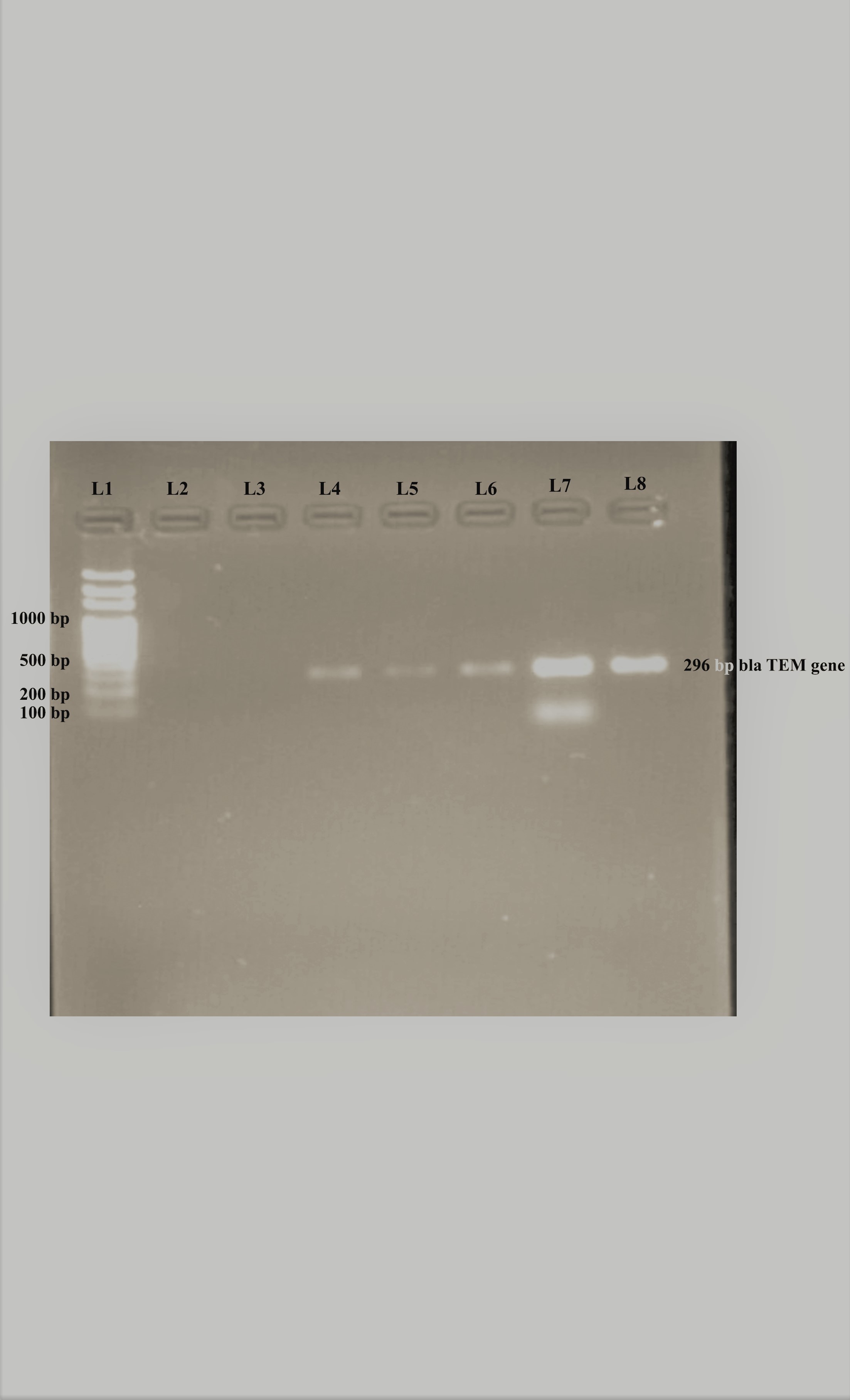

Supplement: Supplementary file 1 — Supplementary Material 1 [file 12941_2023_584_MOESM1_ESM.jpg]

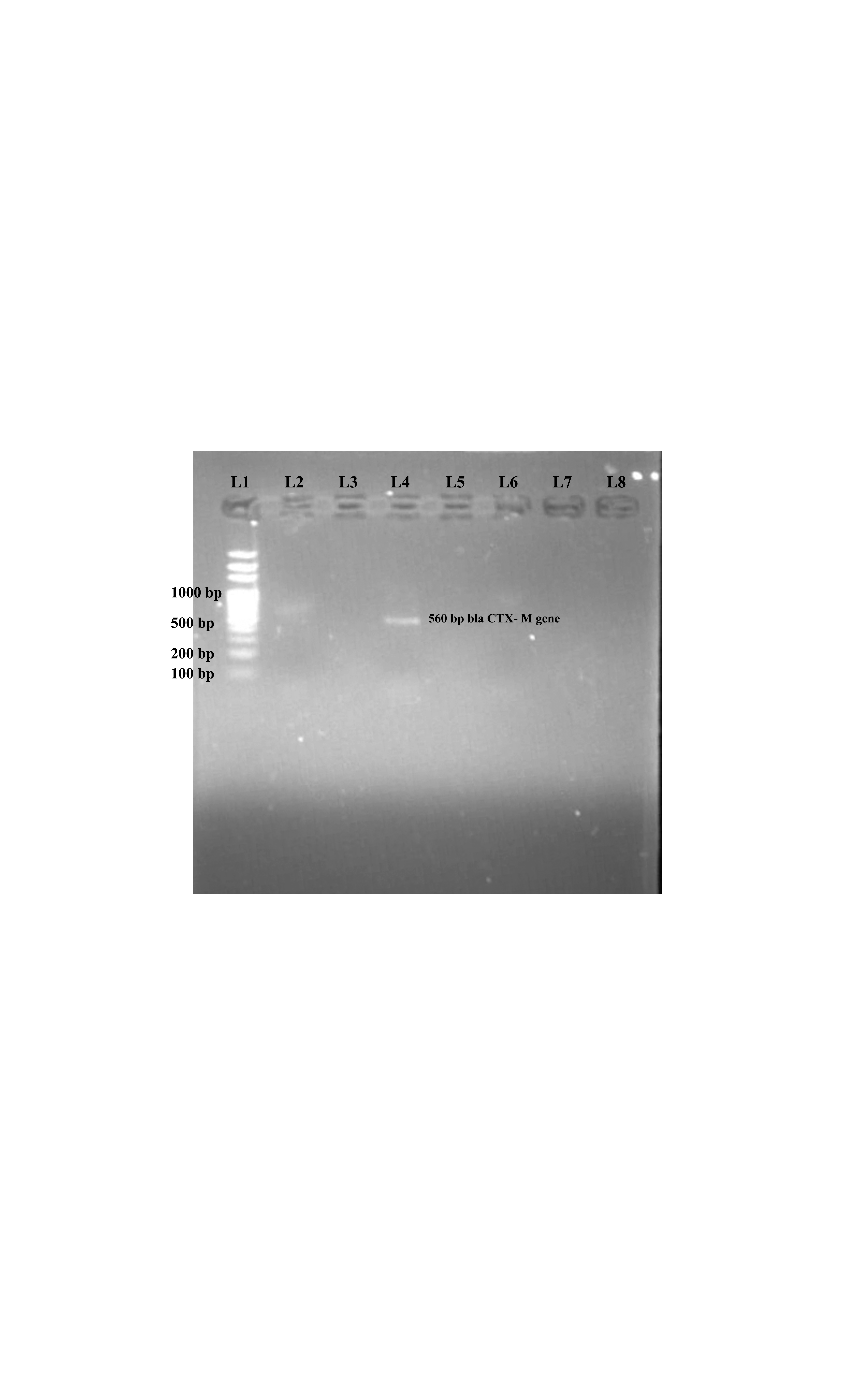

Supplement: Supplementary file 2 — Supplementary Material 2 [file 12941_2023_584_MOESM2_ESM.jpg]
