# Supplementary material for: Molecular detection of plasmid mediated blaTEM, blaCTX−M,and blaSHV genes in Extended Spectrum β-Lactamase (ESBL) Escherichia coli from clinical samples
Source: Ann Clin Microbiol Antimicrob. 2023 May 5;22:33. doi: 10.1186/s12941-023-00584-0 (PMC10163748; doi:10.1186/s12941-023-00584-0)
Supplement: Supplementary file 3 — Supplementary Material 3 [file 12941_2023_584_MOESM3_ESM.docx]

Molecular Detection of Plasmid Mediated *bla*_TEM_, *bla*_CTX-M_ and *bla*_SHV_ Genes in Extended Spectrum β-Lactamase (ESBL) *Escherichia coli* from Clinical Samples

Mahesh Kumar Chaudhary, ^1, 2^ Indrani Jadhav, ^3^ Megha Raj Banjara ^4^

^1^School of Life and Basic Sciences, Jaipur National University,Jaipur,India,

^2^Department of Microbiology, Nepal Mediciti Hospital,Lalitpur,Nepal

^3^School of Life and Basic Sciences, Jaipur National University,Jaipur,India

^4^Central Department of Microbiology,Tribhuvan University,Kirtipur,Nepal

Corresponding author

Mahesh Kumar Chaudhary

Nepal Mediciti,Bhaisepati,Lalitpur

mahesh_3272@yahoo.com

**ABSTRACT**

**Background**: Extended spectrum β-lactamases (ESBLs) are the group of beta-lactamase enzymes which confer resistance to the oxyimino-cephalosporins and monobactams. Emergence of ESBL producing genes possess a serious threat for the treatment of infections since it is found to be associated with multi-drug resistance. This study was focused to identify the ESBLs producing genes from *Escherichia coli* isolates from clinical samples from a referral level tertiary care hospital in Lalitpur.

**Methods**: This was a cross-sectional study conducted during September 2018 to April 2020 at Microbiology Laboratory of Nepal Mediciti Hospital. Clinical samples were processed and culture isolates were identified and characterized following standard microbiological techniques. Antibiotic susceptibility test was performed by modified Kirby-Bauer disc diffusion method as recommended by Clinical and Laboratory Standard Institute guidelines. Extended spectrum beta-lactamases were phenotypically confirmed by combined disc method. ESBL producing genes *bla*_TEM_, *bla*_CTX-M_and*bla*_SHV_ were confirmed by PCR.

**Results**: Of the 1449 total *E. coli* isolates, 323/1449(22.29%) isolates were multi-drug resistant (MDR). Among total MDR *E. coli* isolates, 215/323(66.56%) were ESBL producers. The maximum number of ESBL *E. coli* was isolated from urine 194(90.23%) followed by sputum 12(5.58%), swab 5 (2.32%), pus 2 (0.93%) and blood 2 (0.93%). Antibiotic susceptibility pattern of ESBL *E.coli* producers showed highest sensitivity towards tigecycline (100%) followed by polymyxinb, colistin and meropenem.Out of 215 phenotypically confirmed ESBL *E.coli*, only 186(86.51%) isolates were found to positive by PCR for either *bla*_TEM_ or *bla*_CTX-M_ genes. Among the ESBL genotypes, most common was *bla*_TEM_ 118(63.4%) followed by *bla*_CTX-M_ 68(36.6%).

**Conclusion**: The emergence of MDR and ESBL producing *E.coli* isolates with high antibiotic resistant rates to commonly used antibiotics and increased predominance of major gene types *bla*_TEM_ is a serious concern to the clinicians as well as microbiologist. Periodic monitoring of antibiotic susceptibility and associated genes would help to guide rationale use of antibiotics for the treatment of predominant pathogen *E. coli* in the hospitals and health care facilities of the communities.

**Keywords**: *Escherichia coli*, Extended spectrum β-lactamase, Multi-drug resistance

1. **INTRODUCTION**

Extended-spectrum beta-lactamases (ESBLs) are the group of beta-lactamase enzymes, which hydrolyze and cause resistance to the oxyimino-cephalosporins (cefotaxime,ceftazidime,ceftriaxone,cefuroxime and cefepime) and monobactams(aztreonam), but not the cephamycins (cefoxitin and cefotetan) or carbapenems (imipenem, meropenem, and ertapenem), produced by *Escherichia coli* and *Klebsiella pneumoniae*[1].

Emergence of resistant bacteria worldwide is a threat to favorable outcomes of treatment of common infections in community and hospital settings. *E.coli* is one of the commonest pathogen to exhibit multi-drug resistance. Important risk factors for infection with MDR and ESBL *E. coli* are prolonged antibiotic exposure, overstay in hospital, increased use of third generation cephalosporins, severe illness, increased use of intravenous devices or catheters [2].

ESBL was first detected during 1983-1990 from different countries [3].Distinct epidemic clones with TEM and SHV enzymes have been found in Europe including SHV-12, CTX-M-9, CTX-M-3 andCTX-M-15 [4].

The prevalence of ESBL producing organisms is more than 20% in Asia and South Africa**.**

In Nepal also due to the increasing incidence of ESBL producing *E.coli*, the cost associated with the treatment has increased. The detection of major genes such as *bla*_TEM_, *bla*_CTX-M_ and *bla*_SHV_ in ESBL producing *E.coli* by molecular methods and their antibiotic resistance pattern can provide valuable information about their epidemiology and help in formulation of rational antimicrobial therapy [5].Therefore, this study was conducted with the objectives of determining the spectrum of MDR and ESBL *E. coli* producing strains and molecular characterization of these resistant genes. Characterization of ESBL *E.coli* at molecular level would be useful for the development of better treatment strategy and prevention of the disease.

**2. MATERIALS AND METHODS**

**2.1 Sample Processing and Identification of Bacteria**

A cross sectional study was conducted in Microbiology Laboratory of Nepal Mediciti Hospital, Bhaisepati; Nepal from September 2018 to April 2020. The ethical approval was obtained from the Ethical Review Board of Nepal Health Research Council (NHRC), Kathmandu, Nepal. A total of 16542 clinical samples sent to the microbiology laboratory were processed and cultured following standard microbiological techniques. The identification of bacterial isolates were carried out by cultural and morphological characters, Gram stain and biochemical tests (triple sugar iron, indole, citrate, urease and motility).

**2.2 Antibiotic Susceptibility Tests**

Antibiotic susceptibility testing was performed by modified Kirby-Bauer disc diffusion method as recommended by Clinical and Laboratory Standard Institute guideline**.** The antibiotics used were amikacin (30µg), gentamycin (10µg), ciprofloxacin (30µg), ceftriaxone (30µg), cefotaxime (30µg), ceftazidime (30µg), nitrofurantoin (300µg), norfloxacin (10µg), nalidixicacid (30µg) ofloxacin (5µg), cotrimoxazole (25µg),cefixime (5µg), cefepime (30µg), tigecycline (15µg), imipenem (10µg), meropenem (10µg), polymyxin b (300µg) and colistin (10µg). Plates were incubated aerobically at 37°C for 24 hours. Zone diameter in millimeters was measured and organisms were identified as sensitive, resistant and intermediate as per CLSI 2022 guidelines [6]. *E.coli* strain ATCC 25922 was used as control strain.

**2.3 Screening of ESBL**

The screening was done by disc diffusion technique using 3^rd^ generation cephalosporins (ceftazidime, cefotaxime and ceftriaxone). Isolates resistant to more than one of these agents were identified as possible ESBL producers [6].

**2.4 Confirmation of ESBL**

For confirmation, combined disc test was performed using ceftazidime (30µg) alone and ceftazidime with clavulanic acid (30µg/10µg) and cefotaxime (30µg) and cefotaxime with clavulanic acid (30µg/10µg).A difference in zone of inhibition by ≥5mm of either of ceftazidime clavulanic acid with ceftazidime alone and cefotaxime clavulanic acid with cefotaxime alone was interpreted as confirmed ESBL [6].

**2.5 Gene Detection**

From confirmed ESBL *E.coli*, plasmid DNA was extracted using alkaline hydrolysis method. These plasmid DNA were used as a template for PCR amplification using *bla*_TEM_, *bla*_CTX-M_ and *bla*_SHV_ specific primers(Marcogen,Korea). For PCR amplification, 1.5µl plasmid DNA was added to 25 µl mixture containing 13 µl master mixture (Solis Biodyne,Estonia),10.5µl nuclease free water and 0.5µl each of reverse and forward primers**.**PCR was performed in 5 Prime/02 thermal cycler using optimized condition(Bibby Scientific,U.K.). For *bla*_TEM_ gene identification, initial denaturation at 94^o^C for 5 minutes followed by 30 cycles of each of denaturation (95^o^C for 45 seconds), annealing (50^o^C for 45 seconds), and extension (72^o^C for 30 seconds), and final extension (72^o^C for 10 minutes).For *bla*_SHV_ and *bla*_CTX-M_ genes, initial denaturation at 94^o^C for 5 minutes followed by 30 cycles of each of denaturation at95^o^C for 45 seconds, annealing at 56^o^C for 45 seconds and 62^o^C for 45 seconds respectively, and extension at 72^o^C for 30 seconds, and final extension at 72^o^C for 10 minutes.The amplified product was subjected to gel electrophoresis (2% gel stained with ethidium bromide) at 70V for 45 minutes.DNA ladder of 100bp was used to estimate the molecular weight of amplified products.

**2.6 Control of the Phenotypic Tests and PCR**

For ESBL test, *E. coli* (ATCC 25922), *Klebsiella pneumoniae* (ATCC 700603) were taken as negative control and positive control respectively*.*Confirmed *E. coli* strains harbouring *bla*_TEM_, *bla*_SHV_ and *bla*_CTX-M_ were taken as positive control and nuclease free water as negative control.

**2.7 Statistical Analysis**

Data were entered and percentage calculations were analyzed using Statistical Package for Social Science (SPSS) version 21.

**3. RESULTS**

1449 *E. coli* isolates were recovered from various clinical samples. The highest number of *E. coli* was isolated from urine followed by sputum,swab,pus,blood,fluid,foley’s tip,vaginal swab, catheter tip,BAL,biopsy,bile suction tube,CVP tip,ET tube. Of the 1449 total *E. coli* isolates, 323/1449(22.29%) isolates were multi-drug resistance.Among total MDR *E. coli* isolates, 215/323(66.56%) isolates were ESBL producers. The maximum number of ESBL *E. coli* was isolated from urine 194(90.23%), followed by sputum 12(5.58%), swab 5 (2.32%), pus 2 (0.93%) and blood 2 (0.93%) (Table 1).

Table 1. Distribution of ESBL *E. coli* from clinical samples

| Specimen | No (%) |
| --- | --- |
| Urine | 194(90.23%) |
| Sputum | 12(5.58%) |
| Swab | 5(2.32%) |
| Pus | 2(0.93%) |
| Blood | 2(0.93%) |
| Total | 215(100.0) |

Table 2: Antibiotic susceptibility pattern of ESBL *E. coli*

| Antibiotics | Antibiotic susceptibility of ESBL *E. coli* (n=215) | |
| --- | --- | --- |
|  | Sensitive | Resistant |
| Amikacin(AK) | 197(91.6%) | 18(8.4%) |
| Gentamycin(G) | 180(83.7%) | 35(16.3%) |
| Ciprofloxacin(CIP) | 125(58.2%) | 90(41.8%) |
| Ceftriaxone(CTR) | 0( 0.0) | 215(100%) |
| Cefotaxime(CTX) | 1 (2.7%) | 214(97.3%) |
| Ceftazidime(CAZ) | 0( 0.0) | 215(100%) |
| Nitrofurantion(NIT)* | 182(93.8%) | 12(6.2%) |
| Norfloxacin(NX)* | 109(56.2%) | 85(43.8%) |
| Nalidixic acid(NA)* | 9(4.6%) | 185(95.4%) |
| Ofloxacin(OF)* | 91(46.9%) | 103(53.1%) |
| Tigecycline(TGC) | 215(100%) | 0( 0.0) |
| Imipenem(IPM) | 148(68.8%) | 67(31.2%) |
| Meropenem(MRP) | 194(90.2%) | 21(9.8%) |
| Polymyxin B(PB) | 215(100%) | 0( 0.0) |
| Colistin(CL) | 215(100%) | 0( 0.0) |

* Used in urinary isolates

Antibiotic susceptibility pattern of ESBL *E. coli* producers showed highest sensitivity towards tigecycline (100%) followed by polymyxin b, colistin and meropenem ( Table 2).

Two hundred fifteen ESBL *E.coli* isolates were confirmed by PCR using *bla*_TEM_, *bla*_CTX-M_ and *bla*_SHV_ specific primers. Out of 215 phenotypically confirmed ESBL *E. coli*, only 186(86.51%) isolates were found to positive by PCR (Table 3).The last 29(13.49%) were negative for any of the resistant genes tested. Among the ESBL genotypes, most common was *bla*_TEM_ 118(63.4%) followed by *bla*_CTX-M_ 68(36.6%) ( Figure 1, Figure 2). The co-existence of *bla*_TEM_ and *bla*_CTX-M_ in ESBL producing *E. coli* was 39(20.96%).No ESBL *E. coli* isolates co-harbored *bla*_SHV_ and *bla*_TEM_, *bla*_CTX-M_ and *bla*_SHV_ genes.

Table 3: Distribution of ESBL genotypes in *E. coli*

| ESBL genotypes | Number (%) (n=186) |
| --- | --- |
| *bla*_TEM_ | 118(63.4%) |
| *bla*_CTX-M_ | 68(36.6%) |
| *bla*_TEM_ + *bla*_CTX-M_ | 39(20.96%) |
| *bla*_SHV_ | 0(0.0) |

**
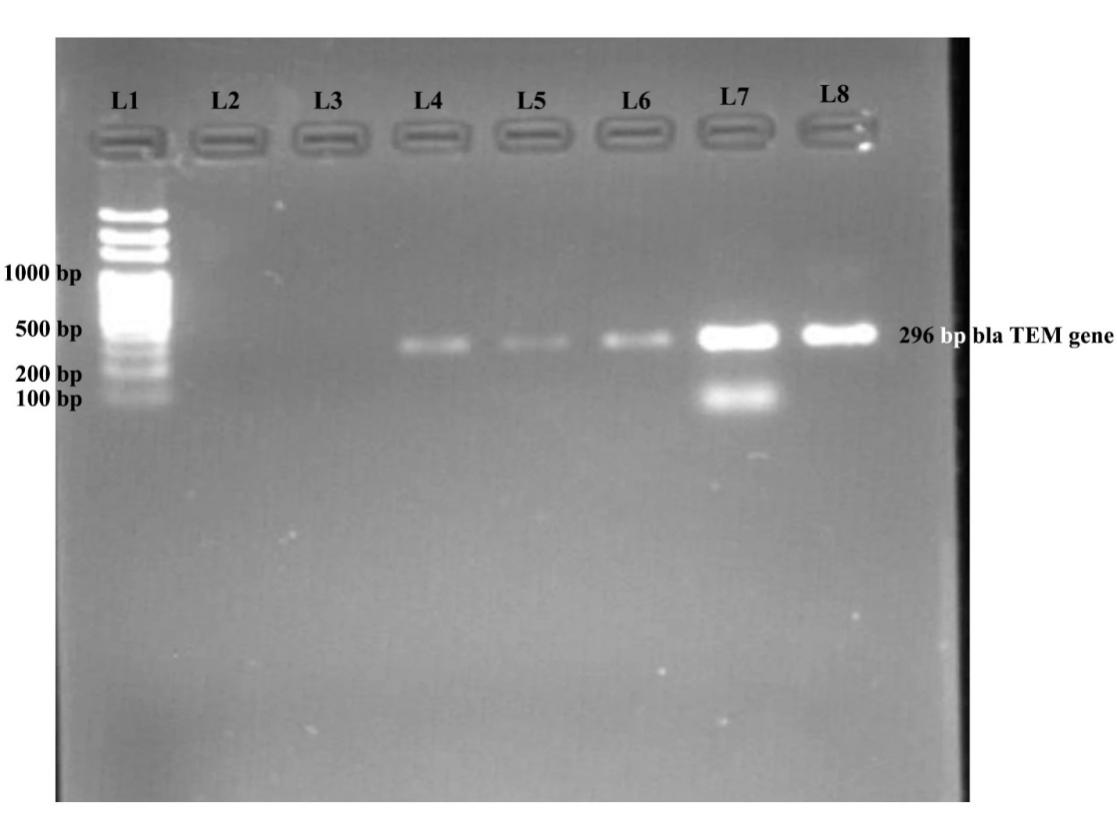
**

Figure 1: Detection of *bla*_TEM_ genes, cropped image of the gel.

**Lane 1:**100 bp DNA ladder. **Lane 2:** Negative control, **Lane 8: Positive control, Lane 4-7:** Test plasmids positive for *bla*_TEM_ gene, **Lane 3:** Test plasmid negative for *bla*_TEM_ gene.

**
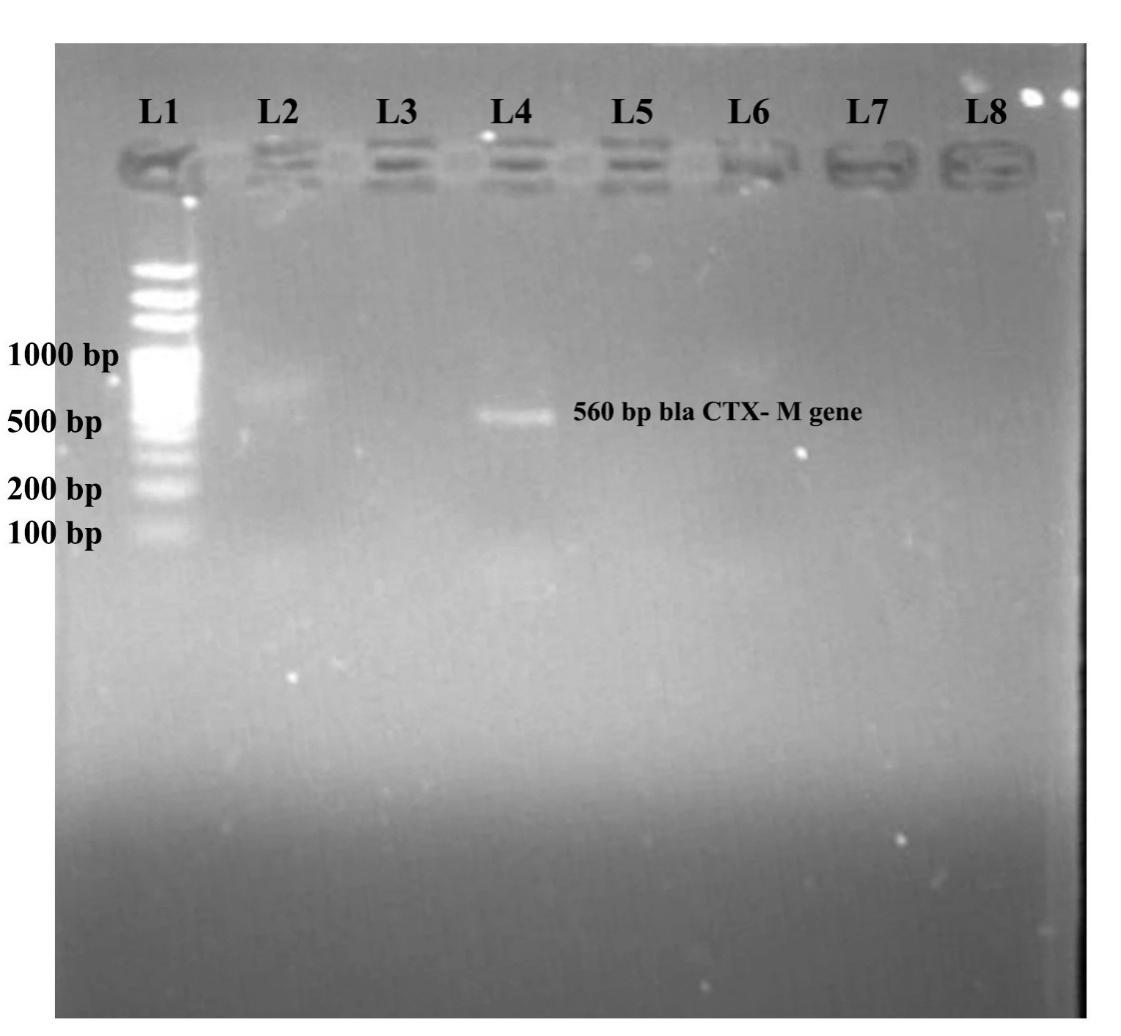
**

Figure 2: Detection of *bla*_CTX-M_ gene, cropped image of the gel

**Lane 1:** 100 bp DNA ladder, **Lane 4: Positive** control, **Lane 3:** Negative control, **Lane 2:** Test plasmid positive for *bla*_CTX-M_ gene, **Lane 5-8:** Test plasmid negative for bla_CTX-M_ gene

**4. DISCUSSION**

Despite the discovery of antibiotics, emergence of MDR and ESBLs producing bacteria due to the extensive use of extended spectrum cephalosporins (ESCs) since early 1980’s is a significant evolution in antimicrobial resistance. Several other factors including misuse of drugs, inappropriate antibiotic treatment, extensive use of antimicrobial has also contributed to the emergence of drug resistant bacteria. The present study was conducted in the department of microbiology laboratory, Nepal Mediciti Hospital during a period of September 2018 to April 2020 with the aim of understanding the antibiotic susceptibility profile of MDR and ESBL producing *E. coli.*

It was found that the highest number of *E. coli* isolates were recovered from urine .With regard to urinary tract infection, *E. coli* showed great extent of resistance to nalidixic acid, co-trimoxazole and third generation cephalosporins. Similar pattern of resistant in urinary isolates of *E. coli* was shown in Nepal [7,8].In contrast to our result, Fanta et al. reported 73% *E. coli* isolates were resistant to ceftriaxone [9].This may be due to the irrational use of third generation cephalosporins [10]. However; a significant degree of susceptibility was found to nitrofurantoin followed by amikacin and gentamycin.Similar findings have been reported in various studies [11,12,13].This may be due to the rational use of these drugs in UTIs cases since it is reserved drug for UTIs.

In this study, analysis of antibiotic susceptibility of *E. coli* isolated from sputum, blood, swab, pus demonstrated a significant degree of susceptibility towards tigecycline (100%) followed by colistin (98% to 100%), polymyxin b (97% to 100%), meropenem (91% to 96%) and imipenem (79% to 90%).Similar results were shown in other studies [14,15].It was found to be higher resistant pattern of cephalosporins(22%to 93%), fluoroquinolones (26% to 85%), aminoglycosides (8% to 59%) as compared to urine isolates. Several studies conducted in Nepal showed similar results [12,16].In contrast to our study, Kubone et al. noted higher susceptibility pattern towards cephalosporins, fluoroquinolones, aminoglycosides [17].The increased level of drug resistance is a major concern worldwide since these are the first line drugs recommended internationally [18]and are irrational used in public and private sectors [19].

The present study noted (323/1449)22.29% MDR *E. coli* isolates that were suspected of being ESBL producers were confirmed by combined disc method. Prevalence of ESBL *E. coli* was (215/323)66.56% which was alarming high. Several studies reported high prevalence i.e.40-70 % of ESBL *E.coli* among MDR *E.coli*[8,13,15,20,21].But the study conducted by Onyedibe et al. in 2018 observed only 18.6% ESBL *E. coli*[22],which is analogous result to other study [23]. This is not similar with our study due to the variation in geography, study design and selection of type of antimicrobial agents. The indiscriminate use of beta-lactam antibiotics leads to the generation of selective pressures which have led to the selection of a variety of mutated forms of beta lactamase [24].Antibiotic profile of ESBL *E. coli* were found to be higher sensitivity towards tigecycline (100%), polymyxin b (100%), colistin(100%) followed by amikacin (91.6%), meropenem (90.2%) and imipenem (68.8%). Susceptibility to nitrofurantoin was 93.8% against ESBL *E. coli* isolated from urine. So, it could be the drug of choice for treating infection caused by ESBL producing *E. coli* similar to the previous studies conducted in India [25,26].

In this study, out of 323 MDR *E. coli* isolates, ESBL *E. coli* phenotypes were found to be positive in 215 (66.56%) isolates. Similar findings were reported by Dalela et al., 2012, Ozcakar et al., 2011 [27,28]. The frequency of phenotypic ESBL positive *E. coli* 29 (13.49%) isolates were lacked *bla*_TEM_, *bla*_CTX-M_ and *bla*_SHV_ genes. Which could be false positive results by phenotypic methods or can be possible presence of other ESBL encoding genes such as SFO, BES, BEL, TLA, GES, PER and VEB types and structural changes in penicillin-binding proteins that result in resistance to β-lactam antibiotics [27,29,30,31].

In this study, the overall prevalence of ESBL genes was 186 (86.51%).Which is similar to other findings reported by Dirar et al, 2020 in Sudan**,** Ahmad etal, 2019 in Iraq [32,33]. PCR analysis revealed the presence of *bla*_TEM_, *bla*_CTX-M_ and *bla*_SHV_ genes in ESBL producing *E. coli* was 118 (63.4%), 68 (36.6%) and 0 (0) respectively. In this study, *bla*_TEM_ was the most predominant genotype of ESBL among *E. coli* isolates. This study is well supported by Dirar et al., 2020 in Sudan, Ahmad et al.,2019 in Iraq, Noha et al., 2020 in Upper Egypt, Pandit et al., 2020 in Nepal, Michael et al., 2018 in Iraq, Sahoo et al. 2019 in India, Jena et al., 2017 in India [32,33,34,35,36,37,38].

In this study, the prevalence of *bla*_CTX-M_ genes were found to be 68 (36.6%) which concurs with various reports demonstrating the extensive worldwide dissemination of *bla*_CTX-M_ genes in ESBL producing *E.coli* isolates [39]. However, another study from Nepal has reported the high prevalence of *bla*_CTX-M_ genes (100%) by Lohani et al., 2019 and (91.4%) by Parajuli et al., 2016 [40,41].

The differences in frequencies of the prevalence of these genes may be as a result of differences in time by which isolates were collected and differences in volume and type of antibiotic consumption [42].

Furthermore, multiple harboring of genes in a single ESBL producing *E.coli* was also noted. The most common combination gene was *bla*_TEM_ + *bla*_CTX-M_ type 39(20.96%).Our finding is in agreement with the study by Lohani et al., where (21.2%) of *bla*_TEM_ and *bla*_CTX-M_ genes were reported [40].The presence of multiple genotypes in a single isolate might be the result of complex antibiotic resistance pattern [43].

Regarding the *bla*_SHV_ gene, no *bla*_SHV_ type *E.coli* was detected in our study, similar to the study in Nigeria [44].However, several findings in Nepal reported the prevalence of *bla*_SHV_ gene in low frequency [40, 41].

**5. CONCLUSION**

This study highlights the emergence of MDR and ESBL producing *E.coli* isolates with high antibiotic resistant rates to commonly used antibiotics and increased predominance of major gene types *bla*_TEM_. No resistance was documented to tigecycline, polymyxin b, and colistin suggesting the suitable drug of choice for treating ESBL producing *E.coli* infections. Periodic molecular detection and identification of ESBL producing bacterial isolates could provide information for rationale use of antibiotics to preserve antibiotics for the future.

**LIST OF ABBREVIATIONS**

ESBL Extended Spectrum Beta Lactamase

MDR Multi Drug Resistant

bla β-lactamase coding gene

ATCC American Type Culture Collection

CLSI Clinical Laboratory Standard Institute

CTX-M Cefotaximase,Munich

TEM Temoniera gene

SHV Sulfhydril variable

**DECLARATIONS**

1. **Ethical approval:** Ethical approval has been attached here with
2. **Consent form:**Consent form to participate has been attached here with
3. **Competing interests**:The authors declare no conflict of interest
4. **Consent for publication**: Not Applicable
5. **Availability of Data and Materials**: All data generated in the study have been included in the manuscript.

**FUNDING**

The authors funded themselves to complete this research

**AUTHORS CONTRIBUTIONS**

Mahesh Chaudhary: Laboratory work, analysis and interpretation of data, drafting manuscript and finalization, final approval to be submitted.

Prof.Dr.Indrani Jadhav:Analysis and interpretation of data,supervision, revision of the manuscript,final approval to be submitted.

Dr.Megha Raj Banjara: Supervision, revision of the manuscript,final approval to be submitted.

**ACKNOWLEDGEMENTS**

We would like to acknowledge Nepal Mediciti Hospital, Nepal for providing ethical approval and research work. We also thank to the microbiology laboratory staffs for their valuable support to complete this work.

**REFERENCES**

**REFERENCES**

1 Ghafourian S, Sadeghifard N, Soheili S, Sekawi Z. Extended Spectrum Beta- lactamases: Definition, Classification and Epidemiology.Curr. Issues Mol. Biol.2015; 17(1):11-22.

<https://doi.org/10.21775/cimb.017.011>

1. Chaudhary U, Aggarwal R. Extended spectrum β-lactamases (ESBL): Emerging threat to

clinical therapeutics. Indian J Med Microbiol.2004;22(2):7.

[https://doi.org/10.1016/S0255-0857(21)02884-X](https://doi.org/10.1016/S0255-0857(21)02884-X)

3 Shaikh S, Fatima J, Shakil S, Mohd S, Rizvi D, Mohammad AK. Antibiotic resistance and extended spectrum beta-lactamases: Types, epidemiology and treatment, Saudi Journal of Biological Sciences.2015; 22(1): 90-101.

<https://doi.org/10.1016/j.sjbs.2014.08.002>

4 Perilli M, Segatore B, Mugnaioli C, Celenza G, Rossolini GM, Stefani S, Luzzaro F,

Pini B, Amicosante G. Persistence of TEM-52/TEM-92 and SHV-12 extended-spectrum beta-lactamases in clinical isolates of Enterobacteriaceae in Italy.Microb. Drug Resist.2011;17(4): 521–524.

<https://doi.org/10.1089/mdr.2011.0059>

5 Kaur M, Aggarwal A.Occurrence of the CTX-M, SHV and the TEM Genes among the Extended Spectrum Beta-Lactamase Producing Isolates of Enterobacteriaceae in a Tertiary Care Hospital of North India. Journal of Clinical and Diagnostic Research.2013;7:642-645.

<https://doi.org/10.7860/JCDR/2013/5081.2872>

6 Clinical and Laboratory Standards Institute (CLSI). Performance Standards for Antimicrobial Susceptibility Testing; 32nded.CLSI supplement M100.Clinical and Laboratory Standards Institute,USA,2022*.*

7 Guragain N, Pradhan A, Dhungel B, Banjara MR, Rijal KR, Ghimire P. Extended Spectrum Beta-lactamase Producing Gram Negative Bacterial Isolates from Urine of Patients Visiting Everest Hospital, Kathmandu, Nepal. Tribhuvan University Journal of Microbiology. 2019;6:26–31.

<https://doi.org/10.3126/tujm.v6i0.26575>

8 Khanal LK, Amatya R, Sah AK, Adhikari RP, Khadka S, Sapkota J, Rai SK. Prevalence of Extended Spectrum Beta Lactamase producing *Escherichia coli* and *Klebsiella* spp. from urinary specimen in a tertiary care hospital. Nepal Medical College Journal.2022; 24(1):75–80.

<https://doi.org/10.3126/nmcj.v24i1.44145>

9 Fanta G, Eshetu M, Mekidim M, Gemechu Z. Antimicrobial Resistance Profile of Different Clinical Isolates against Third-Generation Cephalosporins. Journal of

Pharmaceutics.2018;7:5070742

<https://doi.org/10.1155/2018/5070742>

10 Mitman SL, Amato HK, Saraiva-Garcia C, Loayza F, Salinas L, Kurowski K, et al. Risk factors for third-generation cephalosporin-resistant and extended-spectrum β-

lactamase-producing *Escherichia coli* carriage in domestic animals of semirural parishes east of Quito,Ecuador. PLOS Glob Public Health.2022; 2(3):e0000206.

<https://doi.org/10.1371/journal.pgph.0000206>

11 Salem MA, Ahmed FA. Bacterial Profile of Urinary Tract Infection and Antimicrobial Susceptibility Pattern Among Patients Attending at Bushra Medical Laboratory, Tripoli, Libya. Journal of Gastroenterology and Hepatology Research. 2018; **7(4)**: 2671-2675.

<http://www.ghrnet.org/index.php/joghr/article/view/2380>

12 Subedi S, Chaudhary M, Shrestha B. High MDR AND ESBL Producing *Escherichia coli* and *Klebsiella pneumoniae* from Urine,Pus and Sputum Samples. British Journal of Medicine &Medical Research.2016; 13(10):1-10.

<http://dx.doi.org/10.9734/BJMMR/2016/23350>

1. Rimal U, Thapa S, Maharjan R. Prevalence of Extended Spectrum Beta-Lactamase Producing *Escherichia coli* and *Klebsiella* species from Urinary Specimens of Children attending Friendship International Children’s Hospital.Nepal Journal of Biotechnology.2017;5(1):32-38.

<http://dx.doi.org/10.3126/njb.v5i1.18868>

1. Naqid IA, Balatay AA, Hussein NR, Saeed KA, Ahmed HA, et al. Antibiotic Susceptibility Pattern of *Escherichia coli* Isolated from Various Clinical Samples in Duhok City, Kurdistan Region of Iraq.Int J Infect.2020;7(3):e103740.

<https://dx.doi.org/10.5812/iji.103740>

1. Shilpakar A, Ansari M, Rai KR, Rai G, Rai SK. Prevalence of multidrug-resistant and extended-spectrum beta-lactamase producing Gram-negative isolates from clinical samples in a tertiary care hospital of Nepal. Trop Med Health. 2021;49(1):23.

<https://doi.org/10.1186%2Fs41182-021-00313-3>

16 Yadav K, Prakash S. Screening of ESBL Producing Multidrug Resistant *E.coli* from Urinary Tract Infection Suspected Cases in Southern Terai of Nepal. J Infect Dis Diagn.2017;2:2.

<https://dx.doi.org/10.4172/2576-389x.1000116>

17 Kubone PZ, Mlisana KP, Govinden U, Abia ALK, Essack SY. Antibiotic Susceptibility and Molecular Characterization of Uropathogenic *Escherichia coli* Associated with Community-Acquired Urinary Tract Infections in Urban and Rural Settings in South Africa. Tropical Medicine and Infectious Disease. 2020; 5(4):176.

<https://doi.org/10.3390/tropicalmed5040176>

18 Iftekhar A, Md. Bodiuzzaman R, Sakina S. Antibiotic resistance in Bangladesh: Asystematic review.International Journal of Infectious Diseases.2019;80:54-61.

<https://doi.org/10.1016/j.ijid.2018.12.017>

19 Rijal KR, Banjara MR, Dhungel B, et al. Use of antimicrobials and antimicrobial

resistance in Nepal: a nationwide survey. Sci Rep.2021; 11,11554.

<https://doi.org/10.1038/s41598-021-90812-4>

20 Manandhar S, Zellweger RM, Maharjan N. et al. A high prevalence of multi-drug resistant Gram-negative bacilli in a Nepali tertiary care hospital and associated widespread distribution of Extended-Spectrum Beta-Lactamase (ESBL) and carbapenemase-encoding genes. Ann Clin Microbiol Antimicrob.2020;19:48.

<https://doi.org/10.1186/s12941-020-00390-y>

21 Sadeghi M, Sedigh Ebrahim-Saraie H, Mojtahedi A. Prevalence of ESBL and AmpC genes in *E. coli* isolates from urinary tract infections in the north of Iran. New Microbes New Infect. 2022;45:100947.

<https://doi.org/10.1016%2Fj.nmni.2021.100947>

22 Onyedibe K , Shobowale E, Okolo M, Iroezindu M, Afolaranmi T, Nwaokorie F, Opajobi

S, Isa S, Egah D. Low Prevalence of Carbapenem Resistance in Clinical Isolates of

Extended Spectrum Beta Lactamase (ESBL) Producing *Escherichia coli* in North Central,

Nigeria. Advances in Infectious Diseases.2018; 8:109-120.

<https://doi.org/10.4236/aid.2018.83011>

23 Vachvanichsanong P, McNeil EB, Dissaneewate P. Extended-spectrum beta-

lactamase *Escherichia coli* and *Klebsiella pneumoniae* urinary tract

infections.Epidemiology and Infection.2021;149,e12,1-7.

<https://doi.org/10.1017/S0950268820003015>

# Chanu TR, Shah PK, Soni S, Ghosh A. Phenotypic detection of extended spectrum, AmpC, Metallo beta-lactamases and their coexistence in clinical isolates of commonly isolated gram negative bacteria in GKGH hospital, Bhuj.IP Int J Med Microbiol Trop Dis 2019;5(1):52-56.

<https://doi.org/10.18231/2581-4761.2019.0012>

1. Gharavi MJ, Zarei J, Roshani-Asl P, Yazdanyar Z, Sharif M, Rashidi N. Comprehensive study of antimicrobial susceptibility pattern and extended spectrum beta-lactamase (ESBL) prevalence in bacteria isolated from urine samples. Sci Rep. 2021;11(1):578.

<https://doi.org/10.1038%2Fs41598-020-79791-0>

1. Kumar MS, Arunagirinathan N, Ravikumar M. Antibiotic susceptibility profile of extendedspectrum β-lactamase producing *Escherichia coli,Klebsiella pneumoniae* and *Klebsiella oxytoca* from Urinary tract infections.Research Journal of Pharmacy and Technology.2021;14(8):4425-4428.

<https://doi.org/10.52711/0974-360X.2021.00768>

1. Dalela G, Gupta S, Jain DK, Mehta P. Antibiotic Resistance Pattern in Uropathogens at a Tertiary Care Hospital at Jhalawar with Special Reference to ESBL,AmpC β-

Lactamase and MRSA Production. J Clin D R.2012; 6(4):645-651.

1. Ozcakar ZB,Yalcinkaya F, Kavaz A, Kadioglu G, Elhan AH, Aysev D, Guriz H, Ekim M.Urinary tract infections owing to ESBL-producing bacteria:microorganisms change-

clinical pattern does not.Acta Paediatr.2011;100(8):61-4.

<https://doi.org/10.1111/j.1651-2227.2011.02262.x>

1. Nass T, Poirel L, Nordmann P. Minor extended spectrum β-lactamases.Clin MicrobiolInfect.2008;14 (Suppl.1):42-52.

<https://doi.org/10.1111/j.1469-0691.2007.01861.x>

30 Marthie ME, Chrisna V, Eddy PM, Michael GD, Anwar AH, Marleen MK. Detection of *bla*_SHV_,*bla*_TEM_ and *bla*_CTX-M_ antibiotic resistance genes in randomly selected

bacterial pathogens from the Steve Biko Academic Hospital.Immunol Med

Microbiol.2009;56:191-196.

<https://doi.org/10.1111/j.1574-695x.2009.00564.x>

31 Majid K, Majid B, Fateh R. Detection of TEM,SHV and CTX-M Antibiotic Resistance Genes in *Escherichia coli* Isolates from Infected wounds.Medical Laboratory

Journal. 2017;11(2):30-35.

<https://doi.org/10.18869/acadpub.mlj.11.2.30>

32 Dirar MH, Bilal NE, Ibrahim ME, Hamid ME. Prevalence of extended-spectrum β-

lactamase (ESBL) and molecular detection of *bla*_TEM_, *bla*_SHV_ and *bla*_CTX-M_ genotypes

among *Enterobacteriaceae* isolates from patients in Khartoum, Sudan.Pan Afr Med J.

2020;37:213.

<https://doi.org/10.11604%2Fpamj.2020.37.213.24988>

33 Ahmad Hamad P, Khadija KM. Prevalence of *bla*_TEM_, *bla*_SHV_,and *bla*_CTX-M_

genes among ESBL-producing *Klebsiella pneumoniae* and *Escherichia coli*isolated

from thalassemia in Erbil,Iraq. Mediterranean Journal of Hematology and Infectious

Diseases.2019; 11(1), p. e2019041.

<https://doi.org/10.4084/mjhid.2019.041>

34 Noha AH, Ahmed SK, Eman MF, Adel MH, Medhat AF. Molecular characterization

of Extended-spectrum β lactamase-producing *E.coli*recovered from community-

acquired urinary tract infections in Upper Egypt.Sci Rep.2020;10:2772

<https://doi.org/10.1038/s41598-020-59772-z>

35 Pandit R, Awal B, Shrestha SS, Joshi G, Rijal BP, Parajuli NP. Extended-Spectrum β-

lactamase(ESBL) Genotypes among Multidrug-Resistant Uropathogenic *Escherichia*

*coli* Clinical Isolates from a Teaching Hospital of Nepal. Interdisciplinary Perspectives

on Infectious Diseases.2020;8.

<http://doi.org/10.1155/2020/6525826>

36 Michael NS, Saadi AT. Detection of *bla*_CTX-M_, *bla*_TEM01_ and *bla*_SHV_ Genes in Multidrug Resistant Uropathogenic *E. coli* Isolated from Patients with Recurrent Urinary Tract Infections. International Journal of Medical Research & Health Sciences.2018; 7(9):81- 89.

37 Sahoo S, Otta S, Swain B, Kar SK. Detection and genetic characterization of extended- spectrum beta-lactamases producers in a tertiary care hospital. J Lab Physicians. 2019;11(3):253-258.

<https://doi.org/10.4103/jlp.jlp_31_19>

38 Jena J, Sahoo RK, Debata NK, Subudhi E. Prevalence of TEM, SHV, and CTX-M genes of extended-spectrum beta-lactamases-producing *Escherichia coli* strains isolated from urinary tract infections in adults. Biotech.2017;7(4):244.

<https://doi.org/10.1007/s13205-017-0879-2>

1. Canton R, Gonzalez-Alba JM, Galan JC. CTX-M enzymes:origin and diffusion.Front.Microbiol.2012;3:110.

<https://doi.org/10.3389/fmicb.2012.00110>

40 Lohani B, Thapa M, Sharma L, Adhikari H, Sah AK, Khanal AB, Basnet RB, Aryal M. Predominance of CTX-M Type Extended Spectrum β lactamase (ESBL) Producers Among Clinical Isolates of *Enterobacteriaceae* in a Tertiary Care Hospital, Kathmandu, Nepal. The OpenMicrobiology Journal. 2019; 13:28-33.

<http://dx.doi.org/10.2174/1874285801913010028>

1. Parajuli NP, Maharjan P, Joshi G, Khanal PR. Emerging Perils of Extended Spectrum β-Lactamase Producing Enterobacteriaceae Clinical Isolates in a Teaching Hospital of Nepal. Biomed Research International.2016;7.

<http://dx.doi.org/10.1155/2016/1782835>

42 Al-Agamy HM, Shibl AM, Tawfik AF. Prevalence and molecular characterization of extended-spectrum β-lactamase-producing *Klebsiellapneumoniae* in Riyadh,Saudi Arabia.Annals of Saudi Medicine.2009;29 (4):253.

https://doi.org/10.4103/0256-4947.55306

43 Polse RF, Yousif SY, Assafi MS. Prevalence and molecular characterization of extended spectrum β-lactamases producing uropathogenic *Escherichia coli* isolated in Zakho, Iraq. J Microbiol Infect Dis. 2016; 6(4):163-167.

<https://doi.org/10.5799/jmid.328863>

44 Adekanmbi AO, Akinpelu MO, Olaposi AV, Oyelade AA. Extended spectrum beta-lactamase encoding gene-fingerprints in multidrug resistant *Escherichia coli* isolated from wastewater and sludge of a hospital treatment plant in Nigeria. International Journal of Environmental Studies.2021;78(1):140-150,

<https://doi.org/10.1080/00207233.2020.1778271>
